# Practice patterns and clinical outcomes in acute appendicitis differ in the elderly patient

**DOI:** 10.1007/s00068-024-02620-w

**Published:** 2024-08-10

**Authors:** Matteo Maria Cimino, Alan Biloslavo, Hayato Kurihara, Gabriele Bellio, Matteo Porta, Silvia Fattori, Gary Alan Bass, for the ESTES SnapAppy Group, Gary Alan Bass, Shahin Mohseni, Lewis J. Kaplan, Rebecka Ahl-Hulme, Alan Biloslavo, Yang Cao, Maximilian Peter Forssten, Hayato Kurihara, Isidro Martinez-Casas, Jorge Pereira, Arvid Pourlotfi, Éanna J. Ryan, Matti Tolonen, Nayef Louri, Fatema Nedham, Thomas Noel Walsh, Jamal Hashem, Martin Corbally, Abeer Farhan, Hamad Al Hamad, Rawan Elhennawy, Mariam AlKooheji, Manar AlYusuf, Wissal Aknouche, Anas A. Zeidan, Yusuf S. Alsaffar, Edgar Lipping, Peep Talving, Sten Saar, Katrina Graumann, Liis Kibuspuu, Eduard Harkov, Gisele Aaltonen, Iines S. Sillman, Sami Haapanen, Hanna Lampela, Henna Sammalkorpi, Sofia Eskola, Altti Laakso, Johan Back, Ulla Kettunen, Antti M. Nummi, Anika Szwedyc, Taina Nykänen, Rolle Rantala, Elisa J. Mäkäräinen-Uhlbäck, Sanna A. Meriläinen, Heikki I. Huhta, Jukka M. J. Rintala, Kirsi E. M. Laitakari, Elina Lietzen, Paulina Salminen, Risto K. A. Rapola, Vahid Zangouri, Mohammad Y. Karami, Sedigheh Tahmasebi, Majid Akrami, Alireza Golchini, Faranak Bahrami, Sean M. Johnston, Sean T. Lim, Irele Ifijeh Ahonkhai, Eltahir Eltagani, Odhran K. Ryan, Ailbhe O’Driscoll-Collins, Aine O’Neill, Zakiya Penny, Orlaith Kelly, Carolyn Cullinane, Ian Reynolds, Helen Heneghan, Sean Martin, Des Winter, Matthew Davey, Maha Alkhattab, Aoife J. Lowery, Michael J. Kerin, Aisling M. Hogan, Martin S. Davey, Ke En Oh, Syed Mohammad Umar Kabir, Huilun Huan, Charlotte Aziz, Michael Sugrue, Jessica M. Ryan, Tara M. Connelly, Mohammad Alhazmi, Youssef Al-Mukhaizeem, Fiachra Cooke, Peter M. Neary, Arnold D. K. Hill, Michael R. Boland, Angus J. Lloyd, Frances Fallon, Eoin F. Cleere, James Toale, Patrick A. Boland, Michael Devine, Conor Keady, Sarah Hunter, M. Kevin Barry, Michael E. Kelly, Aidan T. O’Dowling, Ben Creavin, Dara O. Kavanagh, Paul Neary, Paul F. Ridgway, Cathleen A. McCarrick, Jarlath Bolger, Barry Maguire, Cian Keogh, Surbhi Chawla, John Conneely, Emilie McCormack, Ben Shanahan, Nicola Raftery, Darragh Rice, Niall McInerney, Aine Stakelum, Jan Mares, Jonavan Tan, Mark Hanna, Ishwarya Balasubramanian, Christina Fleming, Guy Barsky, Gad Shaked, Simone Giudici, Martina Ceolin, Simona Mei, Francesca Mazzarella, Annalisa Zucca, Susanna Terranova, Nicolo de Manzini, Diego Visconti, Emanuele Doria, Mauro Santarelli, Giovanni Scotton, Francesca Notte, Giacomo Bertelli, Anna Malpaga, Giulia Armatura, Antonio Frena, Dario Tartaglia, Federico Coccolini, Camilla Cremonini, Enrico Cicuttin, Alessio Mazzoni, Massimo Chiarugi, Constança M. Azevedo, Filipa D. Mendes, Luis Q. Faria, Carlos Nazario, Daniela Machado, Miguel Semiao, Jorge Pereira, Carlos Casimiro, Jose Pinto, Tiago Pavão, Raquel Pereira, Bruno Barbosa, Nadia Tenreiro, Catia Ferreira, Goncalo Guidi, Daniela C. Martins, Clara Leal, Bruno B. Vieira, Luís S. Castro, Aldara Faria, Alberto Figueira, Mauro Sousa, Pedro Rodrigues, Rodrigo Roquette, Ricardo Ribeiro, Paulo Cardoso, Joana Domingues, Maria Isabel Manso, Rute Pereira, Tatiana Revez, Bogdan D. Dumbrava, Florin Turcu, Ionut Hutopila, Bogdana Banescu, Gerald Filip, Catalin Copaescu, Marcos Alba Valmorisco, Isabel Manzano Martín, Rocio Martín García de Arboleya, José Ortega Seda, Pablo Rodríguez González, Jose Antonio Becerra Toro, Enrique Rodríguez Lara, Jose Antonio González Minchón, Juan José Segura-Sampedro, Sebastián Jerí-McFarlane, Alejandro Gil-Catalán, Andrea Craus-Miguel, Laura Fernández-Vega, Xavier González-Argenté, Mercedes Estaire-Gómez, Borja Camacho Fernández-Pacheco, Rebeca Vitón-Herrero, Elisa Jimenez-Higuera, Alejandro Barbero, José M. Valverde, Enrique Colás-Ruiz, Maria del Mar Escales-Oliver, Olga Claramonte-Bellmunt, Marta Castro-Suárez, Naila Pagés-Valle, José Andrés Cifuentes-Ródenas, Marta Merayo Alvarez, Jose Luis Michi Campos, Luis Alejandro García González, Beatriz Carrasco Aguilera, Jaime Iturbe Menéndez, Jose Luis Rodicio Miravalles, Carmen Rodríguez Haro, Sara Núñez O’Sullivan, Mariana García Virosta, María Hernández O’Reilly, Izaskun Balciscueta-Coltell, Javier Lorenzo-Perez, Sonia Martinez-Alcaide, Susana Martinez-Ramos, Maria Sebastian-Fuertes, Laura Gomez-Romer, Maria M. Pelloni, Aida Cristina Rahy-Martín, Andrés Felipe Yepes-Cano, Julio Reguera-Rosal, Jose A. Lopez-Ruiz, Beatriz Marenco, Marina Retamar-Gentil, Estela Romero-Vargas, Angeles Gil-Olarte, Aitor Landaluce-Olavarria, Begoña Estraviz-Mateos, Jose-Mario De Francisco-Rios, Aitor Sainz-Lete, Ane Emaldi-Abasolo, Manolo Leon-Valarezo, Claudia C. Lopes Moreira, Aintzane Lizarazu Perez, Araceli Rodriguez Gonzalez, Iñigo Augusto Ponce, Ignacio Maria Goena Iglesias, Cristina González-Prado, Guillermo Cabriada, Beatriz López, Michelle C. Otero, Nerea Muñoz-Plaza, Alberto Palomo, Fernando Mendoza-Moreno, Manuel Díez-Alonso, Francisca García-Moreno-Nisa, Belén Matías-García, Enrique Ovejero-Merino, Ana Quiroga-Valcárcel, Luis Sánchez-Guillén, Inmaculada Oller-Navarro, Álvaro Soler-Silva, Antonio Francisco Sanchís-López, Francisco Blanco-Antona, Luis Muñoz-Bellvis, Jaime López-Sánchez, Sonsoles Garrosa-Muñoz, Beatriz Barón-Salvador, Juan Manuel Nieto-Arranz, Andrea Campos-Serra, Raquel Gràcia-Roman, Anna Muñoz-Campaña, Carla Zerpa-Martin, Andrea Torrecilla-Portoles, Tessa Landa, Virginia Durán Muñoz-Cruzado, Felipe Pareja-Ciuró, Daniel Aparicio-Sánchez, Eduardo Perea del Pozo, Sandra Dios-Barbeito, Carlos García-Sánchez, Antonio Jesús García-Moriana, Victor Turrado-Rodriguez, Roser Termes-Serra, Paula Gonzalez-Atienza, Xavier Morales-Sevillano, Alba Torroella, César Ginestà, Alfredo Escartín, Ferney Gomez, Ana Pinillos, Jaume Ortega, Guillermo Lopez, Eric Gutierrez, Estela Membrilla-Fernandez, Francisco Ocho-Segarra, Ana María González-Castillo, Amalia Pelegrina-Manzano, Juan Guzmán-Ahumada, Juan Jose Sancho-Insenser, María Lourdes García-Jiménez, Laura Castro-Diez, Manuel González-Bermúdez, Mónica Torres-Díaz, Carla Madarro Pena, Angélica Blanco Rodríguez, Dhanisha Trivedi, Souheil Reda, Hans Edvardsson, Lovisa Strömmer, Eva-Corina Caragounis, Karin Sillén, Sofia Warfvinge, Fredrik Bergstedt, Philip Enström, Harald Olsson, Anders Rosemar, Nathalie Young, Agnieszka Popowicz, Johanna Lerström, Johanna Jäderbo, Folke Hammarqvist, Hanna Zacharias, Maria B. Wikström, Anna Stene Hurtsén, Haytham Bayadsi, Emma Jansson, Nils Brunstrom, Ellen B. Malers, Per I. Loftås, Anders Möller, Elena Atanasova, Simone N. Zwicky, Beat Schnüriger, Olga Rutka, Arjun T. Kattakayam, Mushfique Alam, John V. Taylor, Andrei Mihailescu, Eszter T. Karip, Ehtisham Zeb, Adam O’Connor, Goran Pokusevski, Mansoor Khan, Charlotte Florance, Christie Swaminathan, Shameen Jaunoo, Mohammed Sajid, Caoimhe C. Duffy, John Rees, Mark J. Seamon, Niels D. Martin, Ian J. McCurry, Emily A. Vail, Bradford C. Bormann, Daniel C. Cullinane, Jaswin S. Sawhney, Jonathan Dreifus, Forest R. Sheppard, Raul Coimbra, Paul Albini, Sara Edwards

**Affiliations:** 1 Department of Emergency Surgery, Fondazione IRCCS Ca’ Granda Ospedale Maggiore Policlinico, Milan, MI Italy; 2 General Surgery Department, Cattinara University Hospital, Strada Di Fiume 447, 34100 Trieste, TS Italy; 3 Division of Traumatology, Surgical Critical Care & Emergency Surgery, Perelman School of Medicine at the University of Pennsylvania, Philadelphia, PA USA; 4 Penn Center for Perioperative Outcomes Research and Transformation (CPORT), University of Pennsylvania, Philadelphia, PA USA

**Keywords:** Acute appendicitis, Elderly, Observational cohort, Diagnosis, Complications, Treatment outcomes

## Abstract

**Background:**

Appendicitis is the most frequent global abdominal surgical emergency. An ageing population, who often exhibit atypical symptoms and delayed presentations, challenge conventional diagnostic and treatment paradigms.

**Objectives:**

This study aims to delineate disparities in presentation, management, and outcomes between elderly patients and younger adults suffering from acute appendicitis.

**Methods:**

This subgroup analysis forms part of ESTES SnapAppy, a time-bound multi-center prospective, observational cohort study. It includes patients aged 15 years and above who underwent laparoscopic appendectomy during a defined 90-day observational period across multiple centers. Statistical comparisons were performed using appropriate tests with significance set at *p* < 0.05.

**Results:**

The study cohort comprised 521 elderly patients (≥65 years) and 4,092 younger adults (18–64 years). Elderly patients presented later (mean duration of symptoms: 7.88 vs. 3.56 days; *p* < 0.001) and frequently required computed tomography (CT) scans for diagnosis (86.1% vs. 54.0%; *p* < 0.001). The incidence of complicated appendicitis was higher in the elderly (46.7% vs. 20.7%; *p* < 0.001). Delays in surgical intervention were notable in the elderly (85.0% operated within 24 h vs. 88.7%; *p* = 0.018), with longer operative times (71.1 vs. 60.3 min; *p* < 0.001). Postoperative complications were significantly higher in the elderly (27.9% vs. 12.9%; *p* < 0.001), including severe complications (6.9% vs. 2.4%; *p* < 0.001) and prolonged hospital stays (7.9 vs. 3.6 days; *p* < 0.001).

**Conclusions:**

Our findings highlight significant differences in the clinical course and outcomes of acute appendicitis in the elderly compared to younger patients, suggesting a need for age-adapted diagnostic pathways and treatment strategies to improve outcomes in this vulnerable population.

## Introduction

Appendicitis remains the most frequently encountered abdominal surgical emergency worldwide, exhibiting an estimated lifetime prevalence of 6.7% to 8.6% [1–3]. Traditionally, the combination of antimicrobial therapy and surgical source control, specifically appendectomy, constitutes the cornerstone of treatment for most afflicted with acute appendicitis [4]. This approach is underpinned by literature suggesting that recovery from uncomplicated appendicitis typically occurs swiftly, allowing patients to promptly resume their pre-illness activities. However, delayed presentation or interactions with comorbidity may increase the likelihood of complicated appendicitis, the sequelae of which may include diminished autonomy, quality of life, and economic productivity, alongside societal impacts through both direct and indirect healthcare expenditures.

Acute appendicitis displays a predominantly bimodal age distribution, frequently occurring in children and young adults with a second peak in the elderly. The incidence in individuals over 65 years is increasing due to demographic shifts towards an aging population who often present unique diagnostic and treatment challenges; for example, only a quarter exhibit classical symptoms, about one-third seek medical help after considerable delays, and approximately half are accurately diagnosed upon initial hospital admission [5]. Such delays are frequently associated with a greater risk of developing severe complications like abscesses or peritonitis. Consequently, the optimal treatment strategy for acute appendicitis in the elderly remains elusive, and few studies specifically address this concern.

We hypothesize that the postoperative incidence of complications, mortality, and extended hospital stay would be greater in patients aged over 65 following appendectomy, when compared with a younger cohort. This hypothesis was tested through post hoc analysis of the ESTES SnapAppy snapshot audit dataset, a time-constrained, prospective, multicenter observational cohort study which took place in 2020 and 2021. We aim to provide a clearer understanding of the differential impacts and needs dictated by age in the diagnosis and management of acute appendicitis, thereby guiding more tailored and effective treatment modalities.

## Methods

We conducted a prospective, observational, non-randomized multicenter cohort study, using standardized published methodology [6], in line with a pre-specified protocol which was registered with ClinicalTrials.gov (Trial # NCT04365491). The SnapAppy study [7–9] enrolled all consecutive patients admitted with acute appendicitis in a 90-day window between November 1, 2020, and May 28, 2021, and followed those patients for 90 days post-admission (up to August 31, 2021). The study complied with both the Strengthening the Reporting of Observational Studies in Epidemiology (STROBE) guidelines and the Declaration of Helsinki.

### Center eligibility

Centers undertaking adult emergency general surgery were eligible to register to enter patients into the study. No minimum case volume, or center-specific limitations were applied. The study protocol was disseminated to registered members of the European Society of Trauma and Emergency Surgery (ESTES) and through national surgical societies.

### Patient eligibility

All adult patients (over 15 years of age) admitted for acute appendicitis who underwent laparoscopic appendectomy during index admission were included in the current study. While the intention of the study was to capture all patients admitted with acute appendicitis, the uploaded cohort was predominantly patients who had undergone operative intervention (appendectomy), likely due to center-level patient identification through operating room registries. Thus, we decided to exclude patients managed non-operatively from this analysis. Appendicitis was graded using the AAST Anatomic Disease Severity grading system for emergency general surgery that provides a uniform method to assess disease severity for a variety of conditions, including acute appendicitis [10–12]. The grading system uses clinical, radiographic, operative, and pathologic criteria to assign an incrementing ordinal severity score of 1 (mild disease limited to the organ) to 5 (widespread severe disease).

### Data capture

Data were recorded contemporaneously and stored on a secure, user-encrypted online platform (SMARTTrial^®^) without patient-identifiable information. Centers were asked to validate that all eligible patients during the study period had been entered, and to attain > 95% completeness of data field entry prior to final submission. The database was closed for analysis on October 1, 2021. Quality assurance guidance to ensure data fidelity was provided by at least one consultant/attending-level surgeon at each site.

### Outcome measures

The primary outcome measure was any postoperative complication within 30 days. Secondary outcomes were severe complications within 30 days defined as Clavien–Dindo classification grade 3 to 5 (reoperation, reintervention, unplanned admission to intensive care unit, organ support requirement, or death) and length of stay (LOS).

### Statistical analysis

Patients who underwent a laparoscopic appendectomy were included for analysis. Patients were grouped based on age < 65 years vs. ≧65 years. Descriptive results are presented as means and standard deviations (SDs) for continuous, normally distributed variables, medians and interquartile ranges (IQRs) for non-normally distributed continuous variables, as well as counts and percentages for categorical variables. Continuous, normally distributed variables were compared using a Student’s *t* test, while non-normally distributed variables were compared using the Mann–Whitney *U*-test. A Chi Square test or Fisher’s exact test was used for categorical variables, as appropriate. In all analyses, a two-tailed *p* value of less than 0.05 was considered statistically significant. Analyses were conducted with the statistical software R 4.1.1 (R Foundation for Statistical Computing, Vienna, Austria) using the jamovi package. Due to the observational nature of the data, and the associative rather than correlative conclusions drawn, propensity score matching was not employed as part of the statistical analysis strategy.

### Ethical considerations

All participating centers had Institutional Review Board approval or equivalent. No patient consent was sought since the current study was purely observational and did not impact patient care. All data were de-identified when uploaded to the secure study database.

## Results

**Fig. 1:**
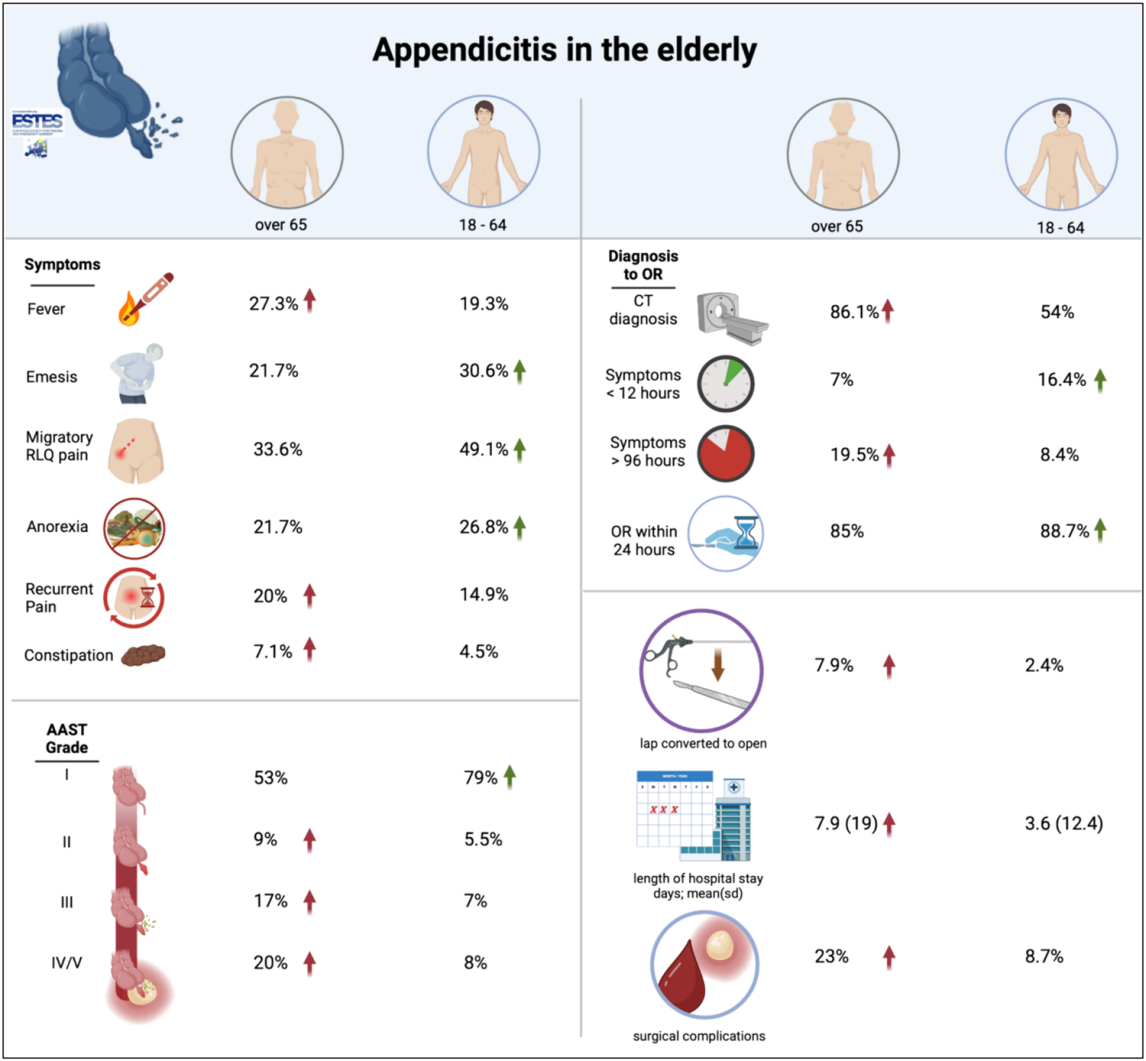
Statistically-significant differences (*p* < 0.005) in presenting symptoms, diagnosis metrics, AAST severity grade and operative metrics between elderly patients and those under the age of 65 years

Our multicenter prospective observational study encompassed a diverse cohort, capturing outcomes for 4,613 patients diagnosed with acute appendicitis, stratified by age (4,092 aged 18–64 vs. 521 aged over 65). The analysis illuminated significant distinctions in the clinical trajectory and outcomes between elderly patients (aged over 65) and the younger demographic (aged 18–64) (Fig. 1). Elderly patients presented later and with less typical symptoms compared to their younger counterparts (Tables 1, 2). The mean duration of symptoms prior to hospital presentation was significantly longer for elderly patients (7.88 days) than for those aged 18–64 (3.56 days), (*p* < 0.001) (Tables 1, 2; Fig. 1). Diagnostic modalities also varied significantly with age; computed tomography (CT) was employed in 86.1% of elderly patients compared to 54.0% of younger patients (*p* < 0.001), reflecting perhaps a need for more definitive imaging given atypical presentations and diagnostic uncertainty in the older group. Serum values of the inflammatory marker C-reactive protein (CRP) were higher in the elderly population than in younger patients, but this was found to be colinear with significantly increasing mean CRP values by AAST severity grade, and thus likely represents the effect of a higher proportion of high-grade disease in the elderly patients.

**Table 1:** Patient demographics, comparison of duration symptoms

	18–64 years *n* = 4092 (88.7%)	Over 65 years *n* = 521 (11.3%)	All patients *n* = 4613	*p*
Sex				
Male	2275 (55.7)	272 (52.2)	2547 (55.3)	0.144
Female	1810 (44.3)	249 (47.8)	2059 (44.7)	
Body Mass Index (BMI)				
Mean (SD)	26.3 (12.3)	28.1 (9.8)	26.5 (12.1)	**0.005**
Method of diagnosis				
CT	2198 (54.0)	446 (86.1)	2644 (57.6)	**<0.001**
Ultrasound	1371 (33.7)	65 (12.5)	1436 (31.3)	
Clinical examination	505 (12.4)	7 (1.4)	512 (11.1)	
Duration of symptoms; *n* (%)				
<12 h	664 (16.4)	36 (7.0)	700 (15.3)	**<0.001**
12–24 h	1329 (32.8)	115 (22.2)	1444 (31.6)	
24–48 h	1031 (25.4)	141 (27.3)	1172 (25.6)	
48–72 h	455 (11.2)	75 (14.5)	530 (11.6)	
72–96 h	236 (5.8)	49 (9.5)	285 (6.2)	
>96 h	341 (8.4)	101 (19.5)	442 (9.7)	
ASA risk classification; *n* (%)				
1	2711 (66.6)	52 (10.1)	2763 (60.3)	**<0.001**
2	1162 (28.6)	239 (46.3)	1401 (30.6)	
3	188 (4.6)	199 (38.6)	387 (8.4)	
4	8 (0.2)	26 (5.0)	34 (0.7)	
Revised Cardiac Risk Index (RCRI); *n* (%)				
1	3989 (97.5)	381 (73.1)	4370 (94.7)	**<0.001**
2	99 (2.4)	114 (21.9)	213 (4.6)	
3	4 (0.1)	21 (4.0)	25 (0.5)	
4	0 (0.0)	5 (1.0)	5 (0.1)

**Table 2:** Presenting symptoms

	18–64 years (*n* = 4092)	Over 65 years (*n* = 521)	*p* value
Pain in RLQ	3462.0 (84.6%)	428.0 (82.1%)	0.147
Migration of pain to RLQ	2010.0 (49.1%)	175.0 (33.6%)	**<0.001**
Recurrent pain	609.0 (14.9%)	104.0 (20.0%)	**0.003**
Anorexia	1096.0 (26.8%)	113.0 (21.7%)	**0.013**
Nausea	1832.0 (44.8%)	189.0 (36.3%)	**<0.001**
Emesis	1251.0 (30.6%)	113.0 (21.7%)	**<0.001**
Fevers	789.0 (19.3%)	142.0 (27.3%)	**<0.001**
Rigors	148.0 (3.6%)	18.0 (3.5%)	0.852
Diarrhea	396.0 (9.7%)	55.0 (10.6%)	0.525
Constipation	183.0 (4.5%)	37.0 (7.1%)	**0.008**

The severity of appendicitis, as graded by the American Association for the Surgery of Trauma (AAST), was higher among the elderly. The proportion of complicated appendicitis (AAST Grade 2 +) was 46.7% in the elderly group compared to 20.7% in the younger cohort (*p* < 0.001). A significant delay in time to the operating room was observed for elderly patients, with a smaller percentage (85.0% compared to 88.7%, *p* = 0.018) being operated on within 24 h of diagnosis. Further, elderly patients underwent laparoscopic procedures less often, with longer median operative times (71.1 min vs. 60.3 min, *p* < 0.001) (Table 3). Surgical drains were significantly more likely to be placed intraoperatively in elderly patients versus those under 65 (136, 30.1% vs. 383, 9.9%. OR = 3.90 (3.08–4.90); *p* < 0.001). Post-operative complications were markedly higher in the elderly, with 27.9% experiencing some form of complication compared to 12.9% of younger patients (*p* < 0.001). Specifically, severe complications classified as Clavien-Dindo Grade 3 + occurred in 6.9% of the elderly compared to 2.4% of younger patients. Post-operative ileus was particularly notable, affecting 8.7% of elderly patients, which starkly contrasts with the 1.8% incidence in the younger population (*p* < 0.001). Post-operative ICU admission, while an infrequent occurrence, was significantly more frequent in the elderly cohort (15, 2.9%) compared with patients under 65 (21, 0.5%); (*p* < 0.001). Hospital stay was also significantly longer in the elderly, with a mean (SD) duration of 7.9 (19) days compared to 3.6 (12.4) days in the younger group (*p* < 0.001) (Table 4). This extended stay was associated with an increased complication rate (*p* < 0.001). Bold values represent those which have achieved statistically-significant differences between groups.

**Table 3:** Time to operating room, operative findings, experience level of operating surgeon

	18–64 years *n* = 4092 (88.7%)	Over 65 years *n* = 521 (11.3%)	*p*
Operated within 24 h of diagnosis; *n* (%)	3629 (88.7)	443 (85.0)	**0.018**
Complicated appendicitis (AAST > 2); *n* (%)	572 (20.7)	225 (46.7)	**<0.001**
AAST			
Grade 1: Acutely inflamed appendix	2190 (79.3)	257 (53.3)	**<0.001**
Grade 2: Gangrenous appendix	153 (5.5)	43 (8.9)	
Grade 3: Perforated appendix with local contamination	193 (7.0)	84 (17.4)	
Grade 4: Perforated appendix with phlegmon/abscess	195 (7.1)	93 (19.3)	
Grade 5: Perforated appendix with Generalised peritonitis	31 (1.1)	5 (1.0)	
Surgical technique			
Laparoscopic	3401 (87.8)	367 (80.5)	**<0.001**
Laparoscopic converted to ope	91 (2.4)	36 (7.9)	
Open	380 (9.8)	53 (11.6)	
Procedure duration (minutes); mean (SD)	60.3 (32.7)	71.1 (36.7)	**<0.001**
Operating surgeon			
Consultant/attending	1231 (31.7)	212 (46.1)	**<0.001**
Resident/fellow	2861 (69.3)	309 (54.9)

**Table 4:** Post-operative complications

	18–64 years *n* = 4092 (88.7%)	Over 65 years *n* = 521 (11.3%)	*p*
Length of hospital stay (days); mean (SD)	3.6 (12.4)	7.9 (19.0)	**<0.001**
Severe complication (Clavien-Dindo 3+	94 (2.4)	34 (6.9)	**<0.001**
Clavien-Dindo complication (grade)			
1	125 (3.2)	43 (8.7)	**<0.001**
2	137 (3.5)	43 (8.7)	
3a	54 (1.4)	15 (3.0)	
3b	37 (0.9)	12 (2.4)	
4a	0 (0.0)	2 (0.4)	
4b	1 (0.0)	0 (0.0)	
5	2 (0.1)	5 (1.0)	
None	3564 (90.9)	375 (75.8)	
Any	356 (8.7)	120 (23)	
Missing	172 (4.2)	26 (4.9)	NS
Re-operation for complications	54 (1.3)	18 (3.6)	**<0.001**
Incisional hernia	11 (0.3)	3 (0.6)	0.425
Wound infection	71 (1.7)	16 (3.1)	0.052
Wound dehiscence	25 (0.6)	5 (1.0)	0.520
Post-operative abscess			
Pelvic	122 (3.0)	24 (4.6)	0.063
Interloop	7 (0.2)	1 (0.2)	1.000
Hemorrhage	9 (0.2)	8 (1.5)	**<0.001**
Post-operative ileus	75 (1.8)	45 (8.7)	**<0.001**

## Discussion

Outcomes following appendectomy differ significantly between elderly patients and younger adults [13]. This is evident not only in the postoperative complication burden, but also in the incidence of mortality and morbidity reported by several studies, even after negative appendectomy [14–16]. Despite the challenges posed by acute appendicitis in the elderly, treatment approaches tailored to patient factors can improve outcomes. Our study supports the hypothesis that there is pronounced variability in presenting symptoms, diagnostic imaging modalities used, and time to the operating room between elderly patients and adults under the age of 65 years. While similar observations have been reported previously, this work is the first and largest to validate this disparity in an observational multicenter, time-bound non-randomized patient series through the snapshot methodology. This approach, which proactively configures and assesses the relevance of included variables prior to data accumulation, guides accurate mapping of knowledge gaps to the granular patient-level data needed to close those gaps [6].

Differential outcomes between elderly patients and younger adults underscore the necessity for adapted treatment strategies throughout all surgical phases. Preoperatively, older patients may benefit from an expedited and assertive diagnostic pathway to counterbalance the reduced diagnostic precision in this group [17]. Our findings align with existing literature, which indicate atypical presenting symptoms and an extended duration from hospital admission to surgery in older patients [18], necessitating heightened diagnostic vigilance and assertive decision-making to mitigate operative delays.

Our data revealed disparities in pre-operative multimorbidity burden between elderly and younger patients. Significantly longer times under general anesthesia, more unplanned post-operative ICU admissions, and a higher likelihood of conversion to open surgery were also seen in the elderly patients. These differences may be influenced by the interaction between pre-existing multimorbidity and appendicitis severity but may also be affected by the upstaging effects of delays in diagnosis (due to atypical symptomatology) and delay to surgical treatment [18, 19]. This tendency towards higher-stage disease can affect surgical decision-making, may necessitate strategies such as the use of staplers and energy devices in practice locations where they are not the default standard [8]. The intraoperative placement of intraabdominal drains, which have been associated with a greater complication burden and lengthier hospital stays in other studies, were also seen with greater frequency in the older cohort.

To our knowledge, this study is the most comprehensive snapshot series comparing elderly and non-elderly patients with acute appendicitis. Our study is limited by constraints in design, center-level approach, and site participation. First, this an observational multicenter nonrandomized study that implies a possible selection bias. The multicenter nature of the study may introduce data imbalance related to center or surgeon-level practice pattern variability. Heterogeneity in diagnostic modalities and practice patterns reflect the real-life non-randomized and broadly inclusive nature of the snapshot study method. The efficacy of non-operative management (NOM) in patients with acute appendicitis could not be assessed due to the low number of patients treated by NOM, likely due to center-level patient identification through operating room registries; thus, the numbers are very small and would be subject to Type I error. While the sample size for NOM in the elderly in our study was insufficient for us to draw conclusions, future study evaluating the effectiveness of NOM in the elderly population may be beneficial. At present, consensus guidance would suggest this approach should be considered only for selected patients because due to the higher incidence of complicated appendicitis increased morbidity in this demographic surgery remains the gold standard treatment [20].

Our study revealed a significantly higher use of CT scanning for diagnosis in the elderly cohort, with the diagnosis frequently established in younger patients through a combination of clinical examination and surgeon-performed point-of-care ultrasound (POCUS). This result may reflect a difference in practice pattern between Europe, where our study was performed, and may not be generalizable to other healthcare systems around the world. While the practice of performing CT scans on most suspected appendicitis cases is standard in the US, it is not always feasible in Europe due to resource constraints and differing clinical practices. In many European centers, particularly those with limited access to out-of-hours CT scan technicians or radiologists, there is a reliance on surgeon-performed point-of-care ultrasound (POCUS) as promoted by the ESTES MUSEC course [21, 22].

Multimorbidity, defined as the presence of combinations of multiple chronic conditions, significantly impacts surgical outcomes, particularly in elderly patients [19]. However, young patients with chronic illnesses, such as those who have survived congenital abnormalities, may also face similar challenges. Our current study primarily focused on age-related differences, but it is plausible that some observations, such as the increased risk of complications and prolonged recovery, apply to younger patients with significant multimorbidity.

Frailty, characterized by decreased physiological reserves and increased vulnerability to stressors, is a critical factor that can influence surgical outcomes independently of multimorbidity. Although our study did not specifically evaluate frailty, it is a recognized confounder in surgical research [23]. Frailty assessments, such as the modified Frailty Index, could provide valuable insights into patient risk profiles and aid in tailoring perioperative care to enhance outcomes. Incorporating frailty evaluations in future studies would help clarify its impact alongside multimorbidity. The reduced inflammatory response in elderly patients, due to immunosenescence, can lead to atypical presentations and delays in seeking medical attention for appendicitis [24, 25].This physiological basis may result in more advanced disease at the time of diagnosis, as observed in our study. Less localization of pain, relapsing remitting symptoms and variability in fever response may contribute to diagnostic challenges and subsequent delays in treatment, which in turn increase the risk of complications. Frailty, immunosenescence, and inflammaging interact synergistically to exacerbate delays in presentation and increase disease severity in elderly appendicitis patients. Frailty decreases physiological reserves, impairing the ability to cope with acute stressors like infections, while immunosenescence reduces the efficiency of the immune response, leading to atypical and less pronounced symptoms. Inflammaging, which refers to the chronic, low-grade inflammation associated with aging, contributes to increased susceptibility to diseases and complications in geriatric patients. In geriatric emergency surgery, this heightened inflammatory state can exacerbate surgical risks, delay recovery, and complicate perioperative management [26].

It is important that we leverage the learnings from this time-bound multi-center prospective observational study in guideline construction to improve treatment strategies, reduce complications, and enhance recovery processes for the elderly. Our study underscores the unique clinical challenges posed by acute appendicitis in the elderly, as evidenced by a non-randomized prospective multicenter study. This evidence underscores the importance of developing age-specific guidelines to enhance outcomes in this patient population. However, variables such as frailty index, rate of relapse, performance status, and cognitive status should be carefully considered before surgical intervention [27–29].Validated preoperative scoring systems may aid clinicians in assessing individual patient risk and facilitating shared decision-making in complex cases [30–32].

## Conclusions

Elderly patients undergoing appendectomy are at greater risk of post-operative complications. We investigated difference in presenting symptoms, pre-hospital duration of symptoms, appendicitis severity grading, prior multimorbidity and post-operative outcomes between elderly patients and adults under 65 in the SnapAppy dataset, which represents the largest available non-randomized, multi-center time-bound prospective observational study of acute appendicitis. Observed delays in presentation, diagnosis and in obtaining surgical source control invite further research to explore the underpinnings of these differences and to formulate effective strategies for their mitigation. The development of age-specific guidelines for the diagnostic and management pathways for suspected acute appendicitis could significantly enhance patient outcomes, particularly in older adults who present unique clinical challenges.

## Data Availability

No datasets were generated or analysed during the current study.

## References

[1] Jones MW, Lopez RA, Deppen JG. Appendicitis. In: StatPearls. Treasure Island: StatPearls; 2023.

[2] Addiss DG, Shaffer N, Fowler BS, Tauxe RV. The epidemiology of appendicitis and appendectomy in the United States. Am J Epidemiol. 1990;132:910–25. 2239906 10.1093/oxfordjournals.aje.a115734

[3] Ferris M, et al. The global incidence of appendicitis: a systematic review of population-based studies. Ann Surg. 2017;266:237–41. 28288060 10.1097/SLA.0000000000002188

[4] Teng TZJ, Thong XR, Lau KY, Balasubramaniam S, Shelat VG. Acute appendicitis-advances and controversies. World J Gastrointest Surg. 2021;13:1293–314. 34950421 10.4240/wjgs.v13.i11.1293 PMC8649565

[5] Storm-Dickerson TL, Horattas MC. What have we learned over the past 20 years about appendicitis in the elderly? Am J Surg. 2003;185:198–201. 12620555 10.1016/s0002-9610(02)01390-9

[6] Bass GA, et al. The snapshot audit methodology: design, implementation and analysis of prospective observational cohort studies in surgery. Eur J Trauma Emerg Surg. 2023;49:5–15. 35840703 10.1007/s00068-022-02045-3 PMC10606835

[7] Bass GA, et al. Clinical practice selectively follows acute appendicitis guidelines. Eur J Trauma Emerg Surg. 2023;49:45–56. 36719428 10.1007/s00068-022-02208-2 PMC9888346

[8] Bass GA, et al. Techniques for mesoappendix transection and appendix resection: insights from the ESTES SnapAppy study. Eur J Trauma Emerg Surg. 2023;49:17–32. 36693948 10.1007/s00068-022-02191-8 PMC9925585

[9] Young N, et al. Graded operative autonomy in emergency appendectomy mirrors case-complexity: surgical training insights from the SnapAppy prospective observational study. Eur J Trauma Emerg Surg. 2023;49:33–44. 36646862 10.1007/s00068-022-02142-3

[10] Crandall ML, et al. Application of a uniform anatomic grading system to measure disease severity in eight emergency general surgical illnesses. J Trauma Acute Care Surg. 2014;77:705–8. 25494421 10.1097/TA.0000000000000444

[11] Shafi S, et al. Measuring anatomic severity of disease in emergency general surgery. J Trauma Acute Care Surg. 2014;76:884–7. 24553565 10.1097/TA.0b013e3182aafdba

[12] Zielinski MD, Guillamondegui O. The acute management of surgical disease. Cham: Springer; 2022.

[13] Weinandt M, Godiris-Petit G, Menegaux F, Chereau N, Lupinacci RM. Appendicitis is a severe disease in elderly patients: a 20-year audit. JSLS. 2020;24:e2020.00046. 32863702 10.4293/JSLS.2020.00046 PMC7444971

[14] Bhullar JS, Chaudhary S, Cozacov Y, Lopez P, Mittal VK. Acute appendicitis in the elderly: diagnosis and management still a challenge. Am Surg. 2014;80:E295–7. 25347482

[15] Blomqvist PG, Andersson RE, Granath F, Lambe MP, Ekbom AR. Mortality after appendectomy in Sweden, 1987–1996. Ann Surg. 2001;233:455–60. 11303128 10.1097/00000658-200104000-00001 PMC1421275

[16] Kraemer M, Franke C, Ohmann C, Yang Q, Acute Abdominal Pain Study Group. Acute appendicitis in late adulthood: incidence, presentation, and outcome. Results of a prospective multicenter acute abdominal pain study and a review of the literature. Langenbecks Arch Surg. 2000;385:470–81. 11131250 10.1007/s004230000165

[17] Körner H, et al. Incidence of acute nonperforated and perforated appendicitis: age-specific and sex-specific analysis. World J Surg. 1997;21:313–7. 9015177 10.1007/s002689900235

[18] Segev L, et al. Acute appendicitis in the elderly in the twenty-first century. J Gastrointest Surg. 2015;19:730–5. 25681217 10.1007/s11605-014-2716-9

[19] Rosen CB, et al. Multimorbidity confers greater risk for older patients in emergency general surgery than the presence of multiple comorbidities: a retrospective observational study. Med Care. 2022;60:616–22. 35640050 10.1097/MLR.0000000000001733 PMC9262850

[20] Di Saverio S, et al. Diagnosis and treatment of acute appendicitis: 2020 update of the WSES Jerusalem guidelines. World J Emerg Surg. 2020;15:27. 32295644 10.1186/s13017-020-00306-3 PMC7386163

[21] Marconi M, et al. Not only FAST The MUSEC^®^ experience in training surgeons. Ann Ital Chir. 2019;90:373–8. 31815729

[22] Zago M, et al. Tailored ultrasound learning for acute care surgeons: a review of the MUSEC (Modular UltraSound ESTES Course) project. Eur J Trauma Emerg Surg. 2016;42:161–8. 27075021 10.1007/s00068-016-0651-z

[23] Hubbard RE, et al. Frailty status at admission to hospital predicts multiple adverse outcomes. Age Ageing. 2017;46:801–6. 28531254 10.1093/ageing/afx081

[24] Ruiz JG, Theou O. Frailty: a multidisciplinary approach to assessment, management, and prevention. Cham: Springer; 2024.

[25] Kumar SJ, Shukla S, Kumar S, Mishra P. Immunosenescence and inflamm-aging: clinical interventions and the potential for reversal of aging. Cureus. 2024;16: e53297. 38435871 10.7759/cureus.53297 PMC10906346

[26] Fulop T, et al. Immunosenescence and inflamm-aging as two sides of the same coin: friends or foes? Front Immunol. 2017;8:1960. 29375577 10.3389/fimmu.2017.01960 PMC5767595

[27] Leiner T, et al. Frailty and emergency surgery: results of a systematic review and meta-analysis. Front Med. 2022;9: 811524. 10.3389/fmed.2022.811524 PMC9008569 35433739

[28] Costa G, et al. The Emergency Surgery frailty index (EmSFI): development and internal validation of a novel simple bedside risk score for elderly patients undergoing emergency surgery. Aging Clin Exp Res. 2021;33:2191–201. 33205380 10.1007/s40520-020-01735-5 PMC8302529

[29] Gottesman D, McIsaac DI. Frailty and emergency surgery: identification and evidence-based care for vulnerable older adults. Anaesthesia. 2022;77:1430–8. 36089855 10.1111/anae.15860

[30] Havens JM, et al. Risk stratification tools in emergency general surgery. Trauma Surg Acute Care Open. 2018;3: e000160. 29766138 10.1136/tsaco-2017-000160 PMC5931296

[31] Bass GA, et al. Cardiac risk stratification in emergency resection for colonic tumours. BJS Open. 2021;5:zrab057. 34228103 10.1093/bjsopen/zrab057 PMC8259498

[32] Bass GA, et al. The revised cardiac risk index is associated with morbidity and mortality independent of injury severity in elderly patients with rib fractures. Injury. 2023;54:56–62. 36402584 10.1016/j.injury.2022.11.039

